# Role of HLA-antigens class 1 in the development of rheumatoid arthritis in Uzbek women

**DOI:** 10.1186/ar3648

**Published:** 2012-02-09

**Authors:** Nazima N Mirakhmedova, Mavlyuda I Mirzakhanova

**Affiliations:** 1 Institute of Immunology of the Academy of Sciences of the Republic of Uzbekistan, Tashkent, Uzbekistan; 2 Tashkent Medical Academy, Tashkent, Uzbekistan

## 

According to the multiple studies women suffer from rheumatoid arthritis (RA) three times more often than men. The women seem to be ill at the age of more active working activity that results in early disability. The great attention is paid to the hereditary factors, particularly, to HLA-system, in the RA development. In this connection the question about early diagnosis and primary prevention of rheumatoid arthritis remain to be important. Consequently, we studied distribution of HLA I class antigens (A, B, C) in 86 Uzbek women with RA. HLA were identified with 2 step standard microlymphocytotoxicity test using antileucocyte HLA-antisera (St-Petersburg, Russia) and rabbit complement. Control group consist of 301 healthy random Uzbeks. In current study 39 antigens were expressed. Higher frequency was found for A25 (15.1% vs. 0.7% in control), A28 (22.1% vs. 4.9%, respectively) with p < 0.001. Antigen A19 (3.5% vs. 11.9% in control, p < 0.01).

In HLA-A locus (17.4% vs. 4.9% in control); B18 were met in 9.3% vs. 3.7% in control, (p > 0.05); B22 (10.5% vs. 1.3% in control, p > 0.05); B27 (15.1% vs. 8.9% in control, p > 0.05).

Cw4 (12.8% vs. 36.2% in control, p > 0.05) met reliably more rare in HLA-A locus.

The highest indicator of risk was established for A25 (RR = 26.6), then for B22 (RR = 8.7), B16 (RR = 4.0), B27 (RR = 2.8), B18 and A10 (RR = 2.7). Results showed that antigens A25 and A28 (p < 0.001), have major effect, while the B16, B18, B22, B27 - additive contribution to the predisposition to the RA among Uzbek women.

Analysis of results in different clinical RA forms revealed association of slowly progressing articular form with antigens: A25 (p < 0.001, RR = 25.2); A28 (p < 0.01, RR = 6.7); whether A10, B16, B27, B22 were not significant (p > 0.05). Fast progressing articular-visceral form development was associated with HLA-A28, A25, B16, B27, and significance of association was established only for A28 (p < 0.001, RR = 7.6). The important moment in our investigation seems to be the association of RA showed unfavorable development in Uzbek women with antigens HLA-B16 which is a split of antigen B8 and antigen B27, being marker of rheumatoid diseases, that correlates with identical research in different populations.

Thus, the results of our investigation show important contribution of HLA in predisposition to rheumatoid arthritis in Uzbek women.

